# Oral Tobacco and Nicotine Marketplace Trends Since the Tobacco Control Act

**DOI:** 10.1001/jamanetworkopen.2025.40747

**Published:** 2025-10-30

**Authors:** Mary Hrywna, Olivia A. Wackowski, Meagan O. Robichaud, Eugene M. Talbot, Patrick V. Barnwell, Cristine D. Delnevo

**Affiliations:** 1 Rutgers Institute for Nicotine & Tobacco Studies, New Brunswick, New Jersey; 2 Department of Health Behavior, Society and Policy, Rutgers School of Public Health, Piscataway, New Jersey; 3 Department of Medicine, Division of General Internal Medicine, Rutgers Robert Wood Johnson Medical School, New Brunswick, New Jersey

## Abstract

This study assesses trends in sales of oral tobacco and nicotine over a 15-year period beginning with the implementation of the Tobacco Control Act in 2009.

## Introduction

The oral tobacco and nicotine marketplace has transformed since the 2009 Tobacco Control Act (TCA) authorized the US Food and Drug Administration (FDA) to regulate tobacco products, including smokeless tobacco (SLT). Following the TCA, SLT products were required to carry larger warning labels, and in 2019 and 2023, 2 SLT brands received modified risk orders permitting their sale with specific health claims. Also, nicotine pouch sales first appeared in retailer scanner data in 2016, quickly gaining 4% of the oral tobacco and nicotine market by 2019 and continuing to grow.^1,2,3^ This segment has seen rapid expansion in brands and flavors.^3,4^ All new products must seek FDA authorization for legal sale in the US, and in January 2025, the first nicotine pouch received such authorization.^5^ Our study analyzes 15 years of US sales data, capturing a key period of regulatory change and product innovation, including brands and flavors.

## Methods

Annual sales data for oral tobacco and nicotine were licensed for convenience stores from the Nielsen company from 2009 to 2024. Nielsen reports unit sales and assigns product attributes for each Universal Product Code, including brand, product, and flavor. Flavors were collapsed into categories outlined in prior research.^6^

Trends were assessed using Joinpoint version 4.9.0 (National Cancer Institute), a segmented regression analysis application, for average annual percentage changes (AAPCs). We present best model fits with each segment described by its short-term trend (annual percentage changes [APCs]). Percentage changes with 95% CIs that did not cross 0 were considered significant (2-sided α < .05). This study was not considered human participant research and exempt from review and consent per the Common Rule. We followed the STROBE reporting guideline.

## Results

The Table summarizes sales by product, brand, and flavor. Oral tobacco and nicotine sales significantly increased from 905.9 million units in 2009 to 1417.8 million units in 2024 (AAPC = 3.0% [95% CI, 2.0% to 3.9%]). Moist snuff sales increased from 2009 to 2011 (APC = 8.1% [95% CI, −2.2% to 19.6%]), plateaued, then significantly declined from 2020 to 2024 (APC = −7.0% [95% CI, −9.9% to −4.0%]) (Figure **A**). While snus sales significantly increased over time (AAPC = 5.5% [95% CI, 3.6% to 7.3%]), they declined sharply from 2018 to 2024 (APC = −7.8% [95% CI, −9.7% to −5.9%]) and market share peaked at 5.3%. Nicotine pouch sales grew considerably from their introduction to 2024 (AAPC = 113.3% [95% CI, 98.3% to 129.4%]), reaching 43.7% of the market by 2024. The initial rapid growth in nicotine pouch sales slowed from 2020 to 2024, but was still significantly increasing (APC = 50.0% [95% CI, 31.3% to 71.4%]).

**Table.** Characteristics of Commercial Oral Tobacco and Nicotine Products Sold in US Convenience Stores, 2009-2024

Characteristic	Units in millions, market share %																AAPC (95% CI)^a^
	2009	2010	2011	2012	2013	2014	2015	2016	2017	2018	2019	2020	2021	2022	2023	2024	
Total market	905.9	1011.1	1058.9	1041.9	1077.0	1087.1	1111.5	1119.6	1108.2	1090.4	1075.3	1235.2	1240.3	1276.1	1352.9	1417.8	3.0 (2.0 to 3.9)^b^
Moist snuff	834.3 (92.1)	928.2 (91.8)	973.1 (91.9)	963.8 (92.5)	997.3 (92.6)	1007.7 (92.7)	1028.1 (92.5)	1036.7 (92.6)	1024.0 (92.4)	1001.0 (91.8)	958.1 (89.1)	1041.3 (84.3)	962.5 (77.6)	908.6 (71.2)	849.6 (62.8)	741.5 (52.3)	−0.6 (−2.1 to 0.9)
Snus	15.4 (1.7)	32.4 (3.2)	39.2 (3.7)	47.9 (4.6)	49.5 (4.6)	50.0 (4.6)	53.4 (4.8)	57.1 (5.1)	57.6 (5.2)	57.8 (5.3)	54.8 (5.1)	51.9 (4.2)	48.4 (3.9)	45.9 (3.6)	41.9 (3.1)	34.0 (2.4)	5.5 (3.6 to 7.3)^b^
Nicotine pouches	NA	NA	NA	NA	NA	NA	NA	NA	3.3 (0.3)	9.8 (0.9)	43.0 (4.0)	113.6 (9.2)	203.4 (16.4)	297.3 (23.3)	437.0 (32.3)	619.6 (43.7)	113.3 (98.3 to 129.4)^b^
Other^c^	56.2 (6.2)	50.6 (5.0)	47.7 (4.5)	30.2 (2.9)	30.2 (2.8)	29.4 (2.7)	30.0 (2.7)	26.9 (2.4)	23.3 (2.1)	21.8 (2.0)	20.4 (1.9)	29.6 (2.4)	26.0 (2.1)	24.2 (1.9)	24.4 (1.8)	22.7 (1.6)	−6.2 (−9.4 to −3.0)^b^
Moist snuff																	
Flavor																	
Mint or menthol	398.0 (47.7)	454.8 (49.0)	488.5 (50.2)	519.5 (53.9)	553.5 (55.5)	581.5 (57.7)	597.3 (58.1)	625.2 (60.3)	623.6 (60.9)	605.6 (60.5)	597.8 (62.4)	651.8 (62.6)	608.3 (63.2)	573.3 (63.1)	525.9 (61.9)	453.1 (61.1)	0.8 (−0.4 to 2.0)
Fruit	55.9 (6.7)	56.6 (6.1)	46.7 (4.8)	35.7 (3.7)	31.9 (3.2)	31.2 (3.1)	32.9 (3.2)	32.1 (3.1)	30.7 (3.0)	34.0 (3.4)	31.6 (3.3)	32.3 (3.1)	26.9 (2.8)	25.4 (2.8)	23.8 (2.8)	18.5 (2.5)	−7.2 (−9.6 to −4.6)^b^
Tobacco	380.5 (45.6)	416.8 (44.9)	437.9 (45.0)	408.6 (42.4)	411.9 (41.3)	395.0 (39.2)	397.9 (38.7)	379.5 (36.6)	369.7 (36.1)	361.4 (36.1)	328.6 (34.3)	357.2 (34.3)	327.2 (34.0)	309.8 (34.1)	299.9 (35.3)	269.2 (36.3)	−2.3 (−3.8 to −0.7)^b^
Brands																	
Copenhagen	184.4 (22.1)	238.5 (25.7)	266.6 (27.4)	292.0 (30.3)	316.1 (31.7)	324.5 (32.2)	334.1 (32.5)	360.8 (34.8)	368.6 (36.0)	373.4 (37.3)	366.9 (38.3)	381.1 (36.6)	353.2 (36.7)	332.5 (36.6)	307.6 (36.2)	271.4 (36.6)	2.4 (1.0 to 3.8)^b^
Grizzly	216.1 (25.9)	240.4 (25.9)	267.6 (27.5)	290.1 (30.1)	313.2 (31.4)	325.5 (32.3)	337.2 (32.8)	335.9 (32.4)	341.0 (33.3)	319.3 (31.9)	313.3 (32.7)	347.8 (33.4)	325.3 (33.8)	296.2 (32.6)	262.5 (30.9)	201.7 (27.2)	−0.5 (−2.0 to 1.1)
Skoal	206.1 (24.7)	222.8 (24.0)	226.7 (23.3)	225.5 (23.4)	215.4 (21.6)	209.6 (20.8)	212.8 (20.7)	199.1 (19.2)	179.2 (17.5)	167.2 (16.7)	148.5 (15.5)	153.1 (14.7)	138.6 (14.4)	126.3 (13.9)	113.0 (13.3)	94.9 (12.8)	−4.0 (−5.1 to −2.8)^b^
Longhorn	40.9 (4.9)	49.2 (5.3)	51.6 (5.3)	39.5 (4.1)	40.9 (4.1)	39.3 (3.9)	40.1 (3.9)	40.4 (3.9)	41.0 (4.0)	42.0 (4.2)	35.4 (3.7)	43.7 (4.2)	41.4 (4.3)	46.3 (5.1)	51.0 (6.0)	51.2 (6.9)	0.9 (−1.1 to 3.0)
Red Seal	45.9 (5.5)	48.3 (5.2)	47.7 (4.9)	37.6 (3.9)	36.9 (3.7)	37.3 (3.7)	36.0 (3.5)	39.4 (3.8)	35.8 (3.5)	34.0 (3.4)	35.4 (3.7)	41.7 (4.0)	36.6 (3.8)	38.2 (4.2)	38.2 (4.5)	36.3 (4.9)	−1.8 (−3.7 to 0)
Timber Wolf	49.2 (5.9)	46.4 (5.0)	39.9 (4.1)	24.1 (2.5)	20.9 (2.1)	19.1 (1.9)	16.5 (1.6)	13.5 (1.3)	11.3 (1.1)	10.0 (1.0)	9.6 (1.0)	13.5 (1.3)	11.5 (1.2)	10.9 (1.2)	10.2 (1.2)	8.9 (1.2)	−4.9 (−6.0 to −3.8)^b^
Kodiak	35.9 (4.3)	37.1 (4.0)	36.0 (3.7)	37.6 (3.9)	35.9 (3.6)	34.3 (3.4)	32.9 (3.2)	31.1 (3.0)	29.7 (2.9)	28.0 (2.8)	26.8 (2.8)	27.1 (2.6)	26.0 (2.7)	25.4 (2.8)	22.9 (2.7)	19.3 (2.6)	−10.3 (−13.0 to −7.5)^b^
All others	55.9 (6.7)	45.5 (4.9)	38.0 (3.9)	17.3 (1.8)	18.0 (1.8)	18.1 (1.8)	18.5 (1.8)	17.6 (1.7)	18.4 (1.8)	27.0 (2.7)	22.0 (2.3)	32.3 (3.1)	29.8 (3.1)	33.6 (3.7)	44.2 (5.2)	58.6 (7.9)	−1.9 (−6.4 to 2.7)
Snus																	
Flavor																	
Mint or menthol	12.2 (79.1)	23.9 (73.8)	30.8 (78.5)	39.1 (81.6)	41.5 (83.7)	42.6 (85.1)	45.8 (85.9)	49.6 (86.8)	50.3 (87.3)	50.6 (87.6)	48.1 (87.7)	45.8 (88.2)	42.6 (88.1)	40.4 (88.0)	36.2 (86.4)	28.6 (84.1)	6.2 (3.8 to 8.7)^b^
Tobacco	3.2 (20.8)	8.4 (26.0)	8.4 (21.5)	8.8 (18.4)	8.1 (16.3)	7.5 (14.9)	7.5 (14.1)	7.5 (13.2)	7.3 (12.7)	7.2 (12.4)	6.7 (12.3)	6.1 (11.8)	5.8 (11.9)	5.5 (12.0)	5.7 (13.6)	5.3 (15.7)	2.2 (0.7 to 3.8)^b^
Brands																	
Camel Snus	14.2 (92.2)	21.5 (66.4)	24.8 (63.4)	39.0 (81.3)	41.3 (83.4)	42.3 (84.5)	45.6 (85.4)	47.7 (83.6)	48.2 (83.6)	46.5 (80.5)	42.8 (78.1)	41.1 (79.2)	38.1 (78.7)	35.8 (78.0)	31.0 (73.8)	24.3 (71.5)	4.3 (1.4 to 7.3)^b^
Marlboro Snus	1.1 (7.2)	10.8 (33.5)	9.5 (24.2)	4.1 (8.5)	2.4 (4.8)	1.4 (2.8)	0.7 (1.4)	0.2 (0.4)	0	0	0	0	0	0	0	0	NR^d^
Skoal Snus	0.1 (0.4)	0	4.8 (12.3)	4.3 (8.9)	3.6 (7.3)	3.4 (6.7)	3.1 (5.8)	4.5 (7.8)	4.4 (7.7)	6.1 (10.6)	7.3 (13.3)	7.4 (14.3)	7.0 (14.5)	7.0 (15.2)	6.3 (15.0)	4.9 (14.5)	37.7 (26.1 to 50.4)^b^
General Snus	0	0	0 (0.1)	0.6 (1.3)	2.2 (4.5)	3.1 (6.1)	3.9 (7.3)	4.7 (8.2)	5.0 (8.7)	5.1 (8.8)	4.7 (8.5)	3.3 (6.3)	3.0 (6.3)	2.6 (5.6)	2.0 (4.8)	2.1 (6.1)	8.1 (−5.0 to 23.0)
Grizzly Snus	0	0	0	0	0	0	0	0	0	0	0	0	0	0.6 (1.2)	2.7 (6.4)	2.7 (7.8)	NR^d^
All others	0 (0.3)	0	0	0	0	0	0.1 (0.1)	0	0	0.1 (0.1)	0.1 (0.1)	0.1 (0.2)	0.2 (0.4)	0	0	0	NR^d^
Nicotine pouches																	
Flavor																	
Mint or menthol	NA	NA	NA	NA	NA	NA	NA	NA	2.6 (78.6)	7.7 (78.7)	33.5 (77.9)	81.3 (71.5)	131.8 (64.8)	189.1 (63.6)	267.0 (61.1)	369.3 (59.6)	106.5 (100.9 to 112.3)^b^
Fruit	NA	NA	NA	NA	NA	NA	NA	NA	0.0 (1.0)	0.1 (1.1)	1.8 (4.3)	11.1 (9.8)	28.9 (14.2)	39.0 (13.1)	52.0 (11.9)	82.4 (13.3)	227.5 (147.6 to 333.2)^b^
Tobacco	NA	NA	NA	NA	NA	NA	NA	NA	0	0	0	3.5 (3.1)	0.2 (0.1)	0	0	0.6 (0.1)	4.0 (−1.6 to 9.9)
Cinnamon	NA	NA	NA	NA	NA	NA	NA	NA	0.5 (15.2)	1.3 (13.7)	4.7 (11.0)	10.0 (8.8)	18.1 (8.9)	25.0 (8.4)	32.3 (7.4)	41.5 (6.7)	92.2 (88.2 to 96.3)^b^
Coffee	NA	NA	NA	NA	NA	NA	NA	NA	0.2 (5.2)	0.6 (6.5)	2.9 (6.8)	6.8 (6.0)	10.2 (5.0)	17.5 (5.9)	26.7 (6.1)	40.9 (6.6)	126.9 (118.3 to 135.8)^b^
Not flavored	NA	NA	NA	NA	NA	NA	NA	NA	0	0	0	0.9 (0.8)	14.4 (7.1)	26.8 (9.0)	59.0 (13.5)	85.5 (13.8)	108.1 (62.2 to 166.9)^b^
Brands																	
Zyn	NA	NA	NA	NA	NA	NA	NA	NA	2.9 (88.6)	9.0 (91.3)	37.0 (86.0)	94.0 (82.7)	134.7 (66.2)	190.3 (64.0)	282.7 (64.7)	392.8 (63.4)	103.4 (99.9 to 107)^b^
On!	NA	NA	NA	NA	NA	NA	NA	NA	0.4 (11.4)	0.7 (7.3)	2.4 (5.5)	9.4 (8.3)	38.4 (18.9)	74.6 (25.1)	114.9 (26.3)	145.0 (23.4)	145.0 (100.7 to 199.2)^b^
Rogue	NA	NA	NA	NA	NA	NA	NA	NA	0	0	0	2.7 (2.4)	11.8 (5.8)	17.2 (5.8)	24.9 (5.7)	46.5 (7.5)	90.2 (31.6 to 174.9)^b^
Velo^e^	NA	NA	NA	NA	NA	NA	NA	NA	0	0.1 (1.4)	3.7 (8.6)	7.5 (6.6)	18.7 (9.2)	14.9 (5.0)	13.5 (3.1)	22.3 (3.6)	41.7 (1.7 to 97.6)^b^
Zone	NA	NA	NA	NA	NA	NA	NA	NA	0	0	0	0	0	0	0	8.7 (1.4)	48.2 (−6.5 to 134.8)
Other	NA	NA	NA	NA	NA	NA	NA	NA	0	0	0	0	0	0.3 (0.1)	0.9 (0.2)	4.3 (0.7)	57.1 (−42.1 to 326.0)

Abbreviations: AAPC, average annual percentage change; NA, not available; NR, not reported.

^a^ AAPC reported for 2009 to 2024, except for nicotine pouches for which the AAPC is reported for 2017 to 2024.

^b^ Statistically significant change (2-sided α < .05).

^c^ Includes largely chew tobacco products but also other oral nicotine products, such as lozenges, tablets, and gum (not including nicotine replacement therapy).

^d^ AAPC not reported due to insufficient data points or product discontinuation.

^e^ In November 2020, the US Business of the British American Tobacco Group acquired the nicotine pouch product assets of Dryft Sciences, LLC and Dryft nicotine pouches were rebranded under the Velo brand name; thus, both brand names are included in these sales.

**Figure.**
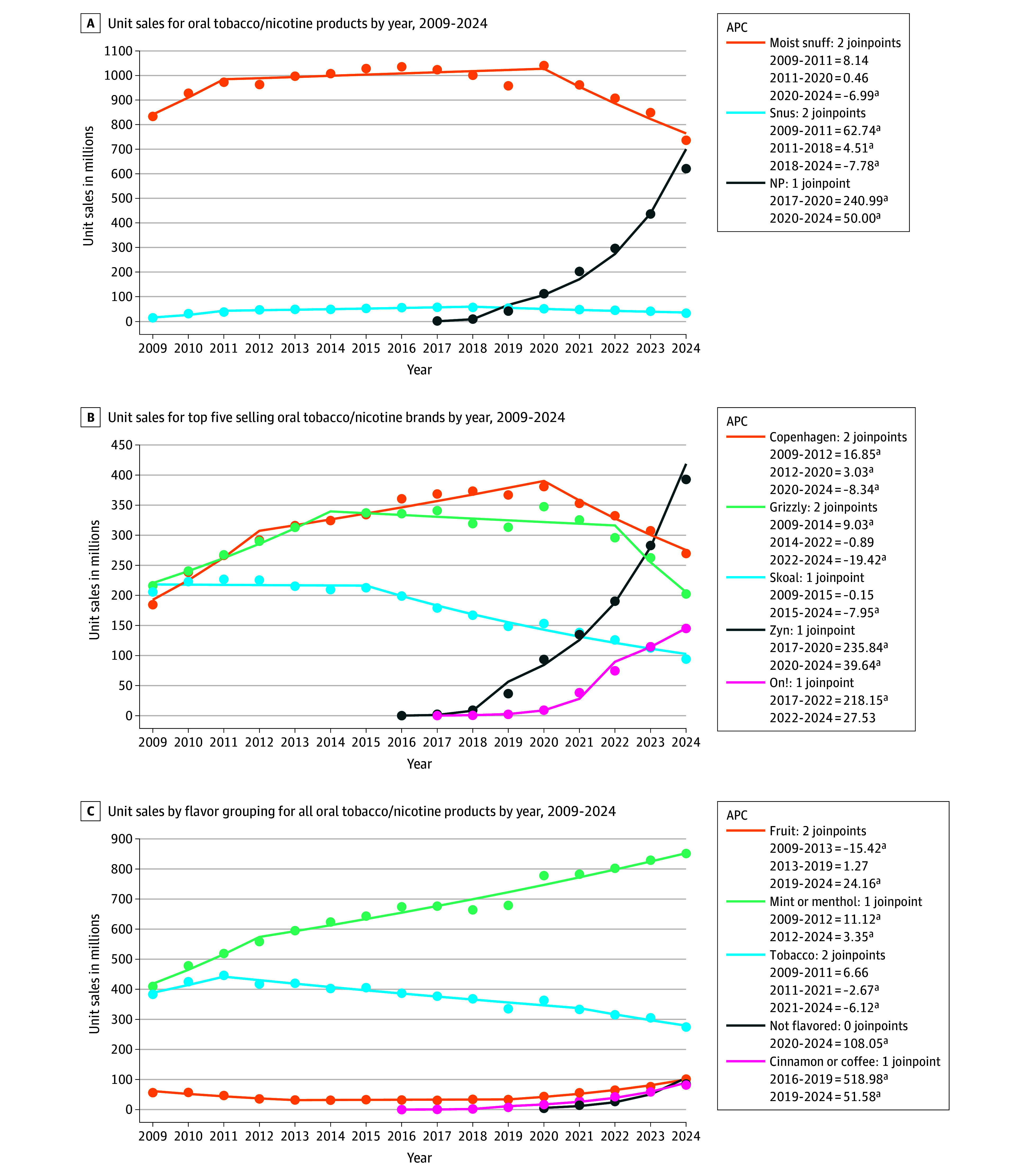
Oral Tobacco and Nicotine Sales Trends Over Time by Product Type, Brand, and Flavor in US Convenience Stores, 2009-2024 APC indicates annual percentage change; NP, nicotine pouch. ^a^Statistically significant change (2-sided α < .05).

From 2009 to 2021, the 3 leading moist snuff brands combined held 66% to 80% market share of the entire oral tobacco and nicotine market, a trend disrupted by nicotine pouches, with Zyn becoming the top selling brand in 2024 (Figure B).

Mint or menthol was the most common flavor for all product types. Nicotine pouches had greater variation in flavors, with fruit flavors gaining market share and “unflavored” nicotine pouches emerging in 2020, accounting for 13.8% of sales in 2024. Across all product types, fruit flavor sales significantly declined from 2009 to 2013 (APC = −15.4% [95% CI, −20.8% to −9.7%]) then increased from 2019 to 2024 (APC = 24.2% [95% CI, 18.6% to 30%]), coinciding with the growth of nicotine pouches (Figure C). Other nontraditional flavors (eg, cinnamon, coffee) also emerged in 2016 and significantly increased.

## Discussion

These data highlight market disruption in the oral tobacco and nicotine category, with considerable declines of SLT products between 2018 and 2024 (including well established brands) coinciding with the growth of nicotine pouches, including nicotine pouch brands becoming market leaders of the entire category.

Study limitations include the inability to account for potential changes in Nielsen’s retailer sample over time and focusing on convenience store sales, which underestimates total sales by excluding other retail channels. The growth of nicotine pouches has important implications as they likely pose lower health risks than combustible cigarettes and SLT given the absence of tobacco, and may serve as potential harm-reduction alternatives, points recognized in the FDA’s 2025 authorization of Zyn.^5^ Continued surveillance of sales and use patterns are needed to understand their public health impact.

## Notes

Supplement.
Data Sharing Statement
