# Supplementary material for: Oral Tobacco and Nicotine Marketplace Trends Since the Tobacco Control Act
Source: JAMA Netw Open. 2025 Oct 30;8(10):e2540747. doi: 10.1001/jamanetworkopen.2025.40747 (PMC12576486; doi:10.1001/jamanetworkopen.2025.40747)
Supplement: Supplement. — Data Sharing Statement [file jamanetwopen-e2540747-s001.pdf]

Data Sharing Statement

Hrywna. Oral Tobacco and Nicotine Marketplace Trends Since the Tobacco Control Act. *JAMA Netw Open*. Published October 30, 2025. doi:10.1001/jamanetworkopen.2025.40747

Data
Data available: No

Additional Information
Explanation for why data not available: Data are proprietary
